# Human Keratoconus Cell Contractility is Mediated by Transforming Growth Factor-Beta Isoforms

**DOI:** 10.3390/jfb6020422

**Published:** 2015-06-18

**Authors:** Desiree’ Lyon, Tina B. McKay, Akhee Sarkar-Nag, Shrestha Priyadarsini, Dimitrios Karamichos

**Affiliations:** 1 Department of Ophthalmology/Dean McGee Eye Institute, University of Oklahoma Health Sciences Center, Oklahoma City, OK 73104, USA; E-Mails: desiree-lyon@ouhsc.edu (D.L.); akhee-sarkernag@ouhsc.edu (A.S.); shrestha-priyadarsini@ouhsc.edu (S.P.); 2 Department of Cell Biology, University of Oklahoma Health Sciences Center, Oklahoma City, OK 73104, USA; E-Mail: tina-mckay@ouhsc.edu

**Keywords:** keratoconus, transforming growth factor-β, collagen gels, extracellular matrix, matrix metalloproteases

## Abstract

Keratoconus (KC) is a progressive disease linked to defects in the structural components of the corneal stroma. The extracellular matrix (ECM) is secreted and assembled by corneal keratocytes and regulated by transforming growth factor-β (TGF-β). We have previously identified alterations in the TGF-β pathway in human keratoconus cells (HKCs) compared to normal corneal fibroblasts (HCFs). In our current study, we seeded HKCs and HCFs in 3D-collagen gels to identify variations in contractility, and expression of matrix metalloproteases (MMPs) by HKCs in response the TGF-β isoforms. HKCs showed delayed contractility with decreased Collagen I:Collagen V ratios. TGF-β1 significantly increased ECM contraction, Collagen I, and Collagen V expression by HKCs. We also found that HKCs have significantly decreased Collagen I:Collagen III ratios suggesting a potential link to altered collagen isoform expression in KC. Our findings show that HKCs have significant variations in collagen secretion in a 3D collagen gel and have delayed contraction of the matrix compared to HCFs. For the first time, we utilize a collagen gel model to characterize the contractility and MMP expression by HKCs that may contribute to the pathobiology of KC.

## 1. Introduction

Keratoconus (KC) is an ecstatic corneal thinning disease that is linked to severe dysfunction in the structural and refractive properties of the cornea [1]. KC affects over 1 in 2000 people worldwide [2]. Age-onset of KC is generally early puberty to middle age and can develop into a progressive disease with detrimental effects on visual acuity [3,4]. Corneal transplantation is the most common option for severe cases [5]. While recent advancements in collagen cross-linking have provided hope for strengthening the KC cornea, its long-term effectiveness and safety has yet to be established [6,7,8]. The molecular pathogenesis of KC is still unclear, and there is currently no animal model for KC. We have previously developed a 3D *in vitro* model of KC disease that mimics the *in vivo* condition [9]. We have shown that human keratoconus cells (HKCs) have an altered phenotype compared to normal human corneal fibroblasts (HCFs) characterized by decreased extracellular matrix (ECM) thickness, increased expression of fibrotic markers, and elevated oxidative stress in a self-assembled 3D-model [9,10]. In our current study, we sought to investigate the HKC disease phenotype in a floating 3D collagen gel matrix in order to measure the contractility of HKCs compared to HCFs, which may provide further insight into molecular defects present in HKCs that give rise to corneal thinning.

Floating 3D collagen gels have been used to study fibrosis and contractility in various cell types, including smooth muscle cells [11], retinal pigment epithelial cells [12], and fibroblasts [13,14,15]. This model utilizes a detached, free-floating collagen gel to mimic the surrounding ECM found in many tissues. Moreover, this 3D model is extremely useful in identifying the role of intracellular defects, such as those observed in HKCs, which may alter the ability of fibroblasts to attach and pull the surrounding collagen ECM. In this model, the cells begin to contract the surrounding ECM, an activity characterized by the formation of stress fibers, which are responsible for the puckering, stretching, and pulling observed when scar formation occurs [16]. Contraction of the ECM by resident cells is required in normal wound healing processes to promote wound closure [17,18]. However, variations in contractility or altered response to growth factors can contribute to development of fibrosis or inability to respond to external stimuli that may delay healing and cause permanent damage to the tissue [19,20]. An altered wound healing response enacted by KC stromal keratocytes in the presence of excessive eye rubbing has been posited to play a role in KC development [21,22,23].

Within the healthy cornea, stromal keratocytes reside natively in an ECM composed primarily of Collagen I (Col I) and Collagen V (Col V) in a ratio of 80:20 along with small glycoproteins and crystallins [24,25,26]. This assembled ECM is important in regulating intracellular processes and provides the structural integrity and refractive power of the cornea [27]. Various studies have identified significant variations in collagen lamellae organization within KC corneal buttons compared to normal controls [28,29]. Furthermore, significant variations in proteoglycan and Col I within KC corneas suggest the presence of deleterious defects in secretion and assembly of the ECM within the stroma that contribute to the KC pathology [30]. Collagen III (Col III) has been found to be upregulated in KC corneal buttons with scarring [31], and we have found that HKCs secrete [32] and assemble [9] higher Col III in a 3D *in vitro* model compared to normal HCFs. Furthermore, a mutation in the Col V locus has been linked to KC development suggesting a potential genetic association between defective collagen assembly and KC [33]. These studies suggest that altered distribution of Col I, III, and V may play an important role in the altered ECM assembled in KC.

TGF-β signaling has been shown to be an important regulator of ECM secretion [34,35], cell differentiation [36,37], and proliferation [38]. There are three primary ligands, TGF-β1, -2, and -3, which are known to modulate downstream genes expression. The pro-fibrotic ligands, TGF-β1 and TGF-β2, activate the canonical TGF-β pathway leading to expression of factors indicative of myofibroblast differentiation, including α-smooth muscle actin (α-SMA) and Collagen III [39,40]. Interestingly, TGF-β3 has been identified as promoting an anti-fibrotic wound healing response with reduced expression of fibrotic markers, but increased native ECM deposition [9,41]. We have previously identified [9,42] significant defects in HKC ECM assembly and the TGF-β pathway, and therefore we sought to investigate the effects of the TGF-β ligands on contractility, collagen deposition, and matrix metalloproteases (MMP) expression by HKCs compared to normal HCFs. To date, this is the first published report using 3D collagen gels to identify novel defects present in HKCs that may contribute to structural defects and corneal thinning.

## 2. Results and Discussion

### 2.1. Contraction Profiles of HCFs and HKCs

**Figure 1:**
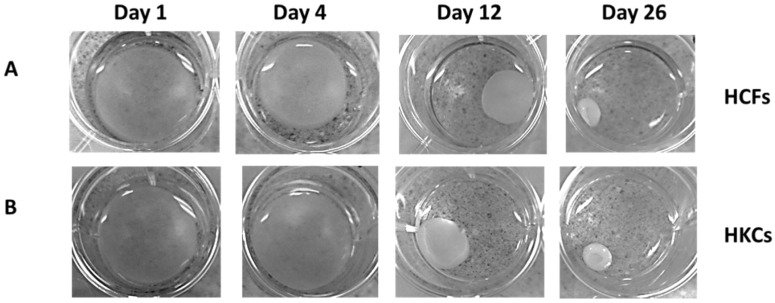
Floating 3D-collagen gel seeded with untreated control (**A**) human corneal fibroblasts (HCFs) and (**B**) human keratoconus cells (HKCs). Change in area of the collagen gel was measured every other day using ImageJ software. Representative images shown, *n* = 3.

**Figure 2:**
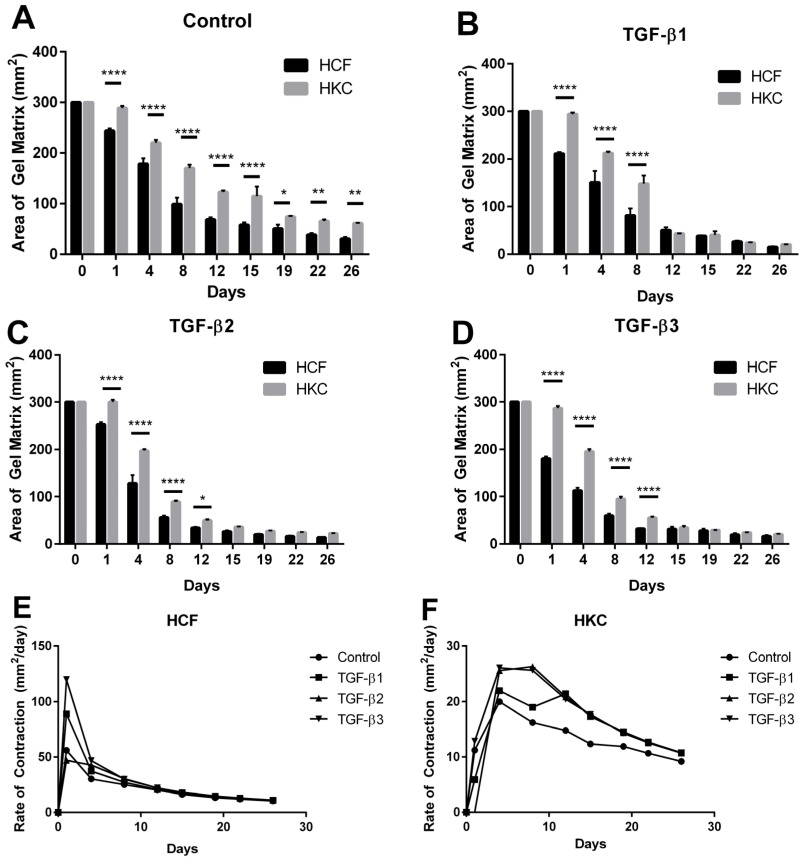
Quantification of the contraction of the collagen matrix in HCFs and HKCs from day 0 to 26. (**A**) control, (**B**) TGF-β1, (**C**) TGF-β2, and (**D**) TGF-β3 samples. A significant reduction in area of the collagen matrix correlates with increased contractility. Rate of contraction from day 0 to day 26 for (**E**) HCFs and (**F**) HKCs. *n* = 3, error bars represent standard error of the mean (SEM). (**** denotes *p* < 0.0001, *** denotes *p* < 0.001, ** denotes *p* < 0.01, and * denotes *p* < 0.05.)

In order to define the role of the surrounding matrix on contractility, we utilized a pre-assembled 3D collagen gel with seeded HKCs and measured rate of contraction compared to normal HCFs. We measured changes in the area of the gel matrix biweekly for 4 weeks in control, TGF-β1, -2, and -3 treated samples using light microscopy, as shown in Figure 1 for representative control samples. At day 1, we identified a 57 mm^2^ (16%) reduction in gel area by HCF controls compared to a 12 mm^2^ reduction (7%) in matrix area in HKCs (Figure 2A, *p* < 0.0001). By day 12, HCFs had contracted the matrix at an average rate of 20 mm^2^/day compared to a contraction rate of 15 mm^3^/day by HKCs (Figure 2E,F). The initial delay in contractility by HKCs corresponded to an incremental delay in shrinkage of the matrix area compared to HCFs, both of which reached maximal contraction by day 26. However, the average rate of contraction from day 0 to day 26 were comparable between HCFs and HKCs (10 mm^2^/day and 9 mm^2^/day, respectively) showing that though HKCs have an initial delayed contractility compared to HCFs, the KC cells eventually reach similar HCF average contraction rate (Figure 2E,F).

In order to identify if HKCs have a differential response to the TGF-β isoforms, we stimulated HCFs and HKCs seeded in the 3D-collagen gels with the three TGF-β isoforms and measured changes in contraction rate. TGF-β1 treatment had an increased effect on contractility in HCFs with a decrease by 89 mm^2^ (32%) in gel area observed from day 0 to day 1 compared to a 6 mm^2^ (2%) reduction by HKCs (Figure 2B, *p* < 0.0001). This delay in contraction was resolved by day 12, at which time, HKCs had contracted to 43 mm^2^, or 15% of the initial area, in the presence of TGF-β1, TGF-β2, or TGF-β3, which was similar to the contraction exhibited by HCFs (Figure 2B–D). Moreover, our results show that TGF-β1 and TGF-β3 stimulate more significant contraction at day 1 with a 89 mm^2^ (32%) reduction in gel size in HCFs compared to a 48 mm^2^ (17%) reduction stimulated by TGF-β2 (Figure 2B–D, *p* < 0.0001). The most significant rate of contraction by HCFs occurred on day 1 following TGF-β1, -2, or -3 stimulation with rates of 88 mm^2^/day, 47 mm^2^/day, and 119 mm^2^/day, respectively (Figure 2E). HKCs exhibited negligible contraction at day 1 but had contraction rates of 22 mm^2^/day, 26 mm^2^/day, and 25 mm^2^/day following stimulation by TGF-β1, -2, and -3, respectively (Figure 2F). This data shows that HKCs have reduced initial contraction, but reach similar contractility by day 12 (20 mm^2^/day) in the presence of TGF-β suggesting increased responsiveness by HKCs to the TGF-β isoforms compared to HCFs. Moreover, TGF-β3 significantly increased the rate of contraction at day 1 (120 mm^2^/day) by HCFs compared to the control (58 mm^2^/day) (Figure 2E). This data suggests that the anti-fibrotic TGF-β3 [43] increases contraction or wound closure by normal stromal fibroblasts and mediates wound healing by directly modulating ECM secretion compared to the fibrotic nature of TGF-β1 and TGF-β2. Further studies are needed to identify the molecular mechanism by which TGF-β3 exhibits anti-fibrotic properties.

Our results show that HKCs have an initial delay in contractility compared to HCFs, which suggests that HKCs are less adept to perform wound closure immediately following injury to the corneal surface. It is well-established that resident cells bind weakly to collagen fibrils directly and instead require linker-proteins, such as fibronectin, to bind to cell-surface integrins and the surrounding collagen bundles [44,45]. TGF-β1 is known to promote expression of both fibronectin [35,46] and integrin subunits important in wound healing [47,48,49]. Our 3D collagen model results show that HKCs are unable to establish initial binding to the collagen gel, but eventually bind and contract the matrix to similar HCF levels by day 26. This data suggests that HKCs may have altered secretion of ECM-linker proteins that delay binding to the pre-assembled ECM. TGF-β1, -2, and -3 stimulation increased the rate of contraction by HKCs and enabled similar contractility to HCFs by day 12, which suggests that TGF-β growth factors stimulate a more contractile-phenotype by HKCs, perhaps by modulating expression of fibronectin and cell-surface integrins.

### 2.2. Collagen Secretion by HCFs and HKCs

**Figure 3:**
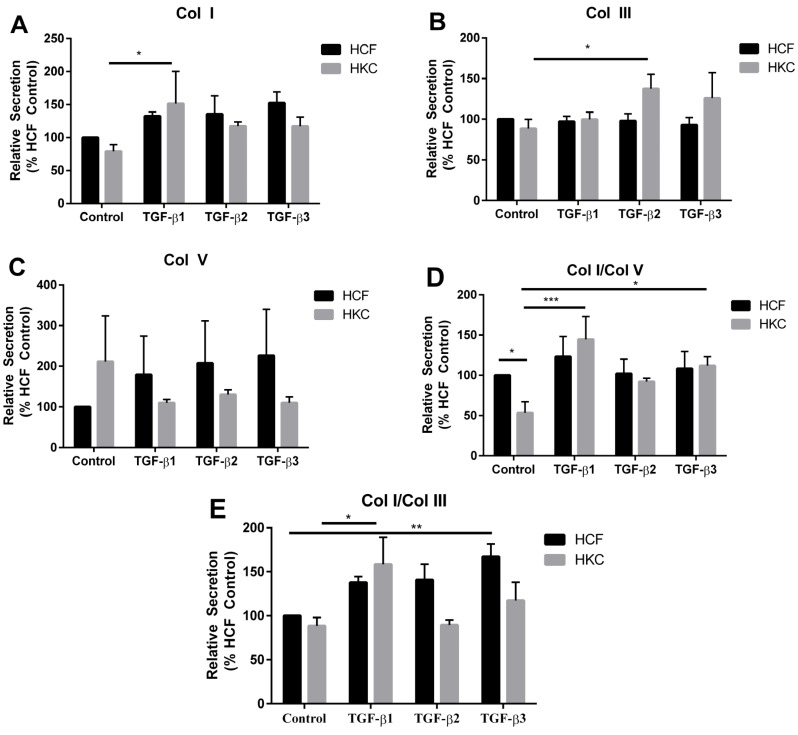
(**A**) Collagen I (Col I), (**B**) Collagen III (Col III), and (**C**) Collagen V (Col V) secretion measured from conditioned media by Western blot from week 1 to week 4. Data reported as ratios of (**D**) Col I/Col V and (**E**) Col I/Col III. *n* = 3. Error bars represent standard error of the mean. (*** denotes *p* < 0.001, ** denotes *p* < 0.01, and * denotes *p* < 0.05.)

Corneal ECM organization and composition provides the structural, mechanical, and physiochemical properties that define the integrity and function of the tissue. KC is characterized by a thin corneal stroma that leads to corneal protrusion and disruption of visual acuity. The major components of the corneal stroma include collagen fibrils and the resident cell, corneal keratocytes, which secrete and assemble the surrounding matrix. The TGF-β pathway is a primary regulator of ECM production by stromal keratocytes. Several studies have identified significant defects in TGF-β signaling and ECM composition [9,43,50,51]. Col I is the dominant structural component of the corneal stroma [52]. Col V is a known regulator of collagen fibrillogenesis and is present at 20% of total collagen composition within the cornea [26,53], whereas Col III is not normally expressed in the uninjured cornea [54,55]. In order to determine the effect of the 3D-collagen gel on ECM secretion by HCFs and HKCs, we measured the amount of Col I, Col III, and Col V secreted into the media by HCFs and HKCs (Figure 3A–C). Basal secretion of Col I was reduced in HKCs by 12% compared to HCFs (Figure 3A). TGF-β1, -2, and -3 increased Col I secretion by 32%, 35%, and 52%, respectively, in HCFs, compared to an increase of 51%, 17%, and 17% by HKCs, respectively (Figure 3A, *p* < 0.05). Col III secretion did not increase significantly in HCFs following treatment with the TGF-β isoform, while HKCs showed increased Col III secretion by 49% following TGF-β2 stimulation (Figure 3B, *p* < 0.05). Col V secretion was not significantly different between the two cell types with or without TGF-β treatment suggesting a significant role for Col I and III regulation between HCFs and HKCs (Figure 3C).

Since the composition of the stromal ECM is tightly regulated and ultimately defines the structural integrity of the cornea, we measured the effect on Col I and Col V ratios. Our results show that Col I/Col V is 47% lower in control HKCs compared to HCFs (Figure 3D, *p* < 0.05). TGF-β1 treatment significantly increased this ratio by 91% in HKCs (Figure 3D, *p* < 0.001). We found that TGF-β2 and TGF-β3 treatment increased the Col I/Col V ratio in HKCs, but not HCFs, by 39% and 59%, respectively. This data shows that the TGF-β isoforms mediate increased Col I/Col V secretion by HKCs suggesting that secretion of select collagen types are regulated by TGF-β signaling, which may play an important role in the wound healing response within the KC cornea. We also measured the effect of the TGF-β isoforms on Col I/Col III secretion in both cell types. TGF-β1 and TGF-β2 treatment did not significantly increase the Col I/Col III ratio in HCFs (Figure 3E). However, TGF-β3 treatment increased this ratio by 67% (Figure 3E, *p* < 0.01) suggesting that TGF-β3 is a potent regulator of expression of specific collagen isoforms by normal HCFs. Col I/Col III increased significantly in HKCs following TGF-β1 stimulation by 70% with a lack of change in this ratio with TGF-β2 or -3 treatment (Figure 3E, *p* < 0.05).

Alterations in ratios of collagen isoforms from normal distributions are known to contribute to corneal dystrophies [31,56]. We have previously shown that HKCs synthesize a significantly thinner ECM compared to normal HCFs [9]. In the collagen gel, seeded HKCs secrete lower Col I/Col V, of which Col V is known to be essential for lamellae formation within the cornea [57], suggesting that collagen fibrillogenesis may be directly modulated in KC. We found that TGF-β isoform treatments increased the basal Col I/Col V ratio to HCF levels, suggesting that modulating TGF-β signaling may alter ECM secretion in KC.

### 2.3. mRNA Expression of Collagen I, III, and V by HCFs and HKCs

**Figure 4:**
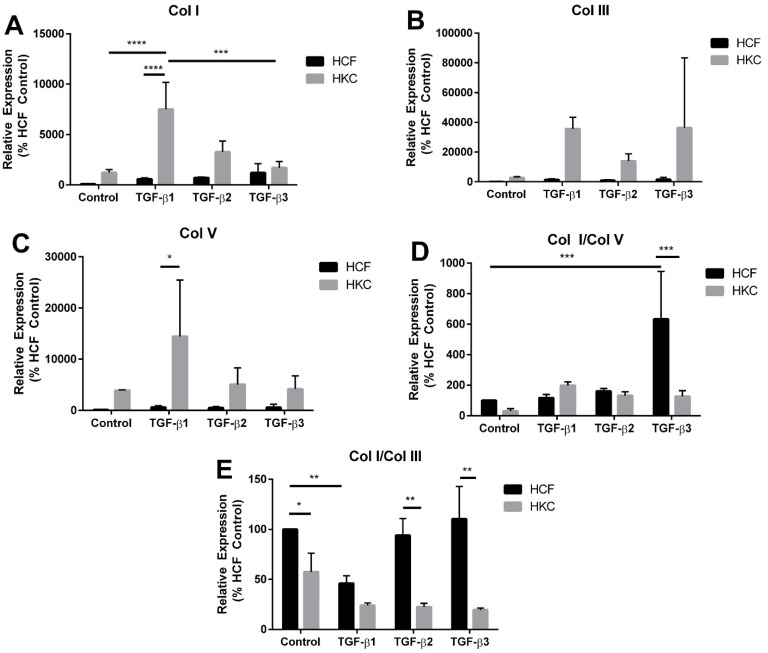
(**A**) Collagen I (Col I), (**B**) Collagen III (Col III), and (**C**) Collagen V (Col V) expression and (**D**–**E**) ratios of Col I/Col V and Col I/Col III by HCFs and HKCs at week 4 measured by RT-PCR. *n* = 3, error bars represent standard error of the mean. (**** denotes *p* < 0.0001, *** denotes *p* < 0.001, ** denotes *p* < 0.01 and * denotes *p* < 0.05.)

In order to determine if expression of pro-collagens correlated with collagen secretion detected in the conditioned media, we quantified the expression of Col I, Col III, and Col V, using RT-PCR in HCF and HKC at day 26 following complete contraction of the matrix. We found an increase in all three collagen types by control HKCs compared to HCFs (Figure 4A–C). We also identified a significant increase of 638% and 994% in expression of Col I and Col III, respectively, by HKCs following TGF-β1 stimulation (Figure 4A,B, *p* < 0.05). In contrast, HCFs did not significantly increase Col I, Col III, or Col V expression following stimulation with TGF-β isoforms (Figure 4A–C). Our results show that HKCs are more responsive to TGF-β isoform treatment compared to HCFs at day 26. This data suggests that fully contracted HCFs have reduced expression of ECM components compared to HKCs.

We also quantified the ratios of collagens expressed in the fully contracted ECM. At day 26, HKCs had increased Col III and Col V expression compared to Col I (Figure 4A–C), which is the dominant collagen isoform produced by normal HCFs. We measured similar Col I/Col V by HCFs and HKCs following full contraction (Figure 4D). Interestingly, we found a substantial increase in Col I/Col V ratio by HCFs following TGF-β3 stimulation. Both TGF-β1 and TGF-β3 increased the Col I/Col V ratio in HKCs by 100%, whereas TGF-β3 increased Col I/Col V secretion by 600% in HCFs (Figure 4D, *p* < 0.01). Col I/Col III ratio was significantly reduced by >50% in HKCs in the presence and absence of the TGF-β isoforms (Figure 4E, *p* < 0.05). A significant downregulation of Col I/Col III expression was noted, by 45% in control HKCs compared to HCFs (Figure 4E, *p* < 0.05). Moreover, we found significant downregulation of Col I/Col III secretion in both cell types following TGF-β1 stimulation supporting the conclusion that TGF-β1 acts as a pro-fibrotic ligand within the corneal stroma. These results show that the Col I/Col III ratio expressed by HKCs in the fully contracted matrix is significantly lower than that of the normal HCFs.

Changes in the ratios of specific collagen types can affect the structural integrity of the ECM and contribute to pathological defects in tissue structure, such as those observed in KC. We found a significant reduction in Col I/Col III ratio by HKCs compared to HCFs. This data supports earlier findings that HKCs have a myofibroblast phenotype that promotes altered ECM structure [9,43]. We have identified that HKCs have defective TGF-β signaling that contributes to expression of pro-fibrotic markers [43]. Our results in this study show that HKCs have increased responsiveness to TGF-β1, -2, and -3 stimulation with increased contractility and Col I/Col V ratios, which alters the native composition and assembly of the surrounding matrix. Since Col V is known to be important in collagen fibrillogenesis [26,53] variations in its expression would be expected to directly affect lamellae assembly. The aberrant expression of Col I/Col V and Col I/Col III in HKCs may be a source of pathogenesis and should be explored further to identify the effects on structural integrity of the KC stroma.

### 2.4. MMP1 and MMP3 Expression by HCFs and HKCs

**Figure 5:**
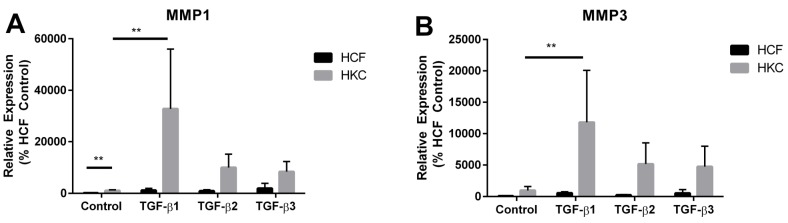
(**A**) MMP1 and (**B**) MMP3 expression in HCFs and HKCs measured by RT-PCR at week 4. *n* = 3, error bars represent standard error of the mean. (** denotes *p* < 0.01.)

MMPs are important in ECM degradation and remodeling within tissues [58]. Increased MMP activity has been posited to play a role in KC disease progression [59,60]. Previous studies have linked upregulation of MMP1 in KC corneal buttons suggesting that degradation of the resident stromal collagen may contribute to KC pathogenesis [61,62]. Furthermore, MMP1 gene expression is transcriptionally regulated with MMP3 gene expression [63], which has yet to be linked to KC. Since KC is associated with thinning of the corneal stroma, we measured expression of MMP1 and MMP3, which are important mediators of ECM degradation in tissues [58]. Interestingly, we measured a 10-fold increase of MMP1 expression in HKCs compared to HCFs (Figure 5A, *p* < 0.01). There was no significant difference in basal expression of MMP3 between HCFs and HKCs (Figure 5B). We found a significant increase in MMP1 expression by over 10-fold with TGF-β1 treatment in both HCFs and HKCs (Figure 5A, *p* < 0.01). TGF-β2 and TGF-β3 increased MMP1 expression by 9-fold and 19-fold, respectively, in HCFs compared to a 2.4-fold and 5.3-fold increase in HKCs (Figure 5A). MMP3 expression also increased in both cell types with TGF-β1, -2, and -3 isoform treatment with a 5.5-fold, 2.4-fold, 5.3-fold, respectively, in HCFs and 8-fold, 4.4-fold, 5.9-fold, respectively, increase in HKCs (Figure 5B).

We measured a significant increase in MMP1 expression, which agreed with earlier reports showing upregulation of MMP1 in corneal buttons [61,62]. Basal expression of MMP3 was not significantly different between HKCs and HCFs, which suggests that MMP3 does not play a prominent role in KC pathogenesis. We measured a significant increase in MMP1 and MMP3 expression in both cell types following stimulation with the TGF-β isoforms. Furthermore, the TGF-β isoforms regulate MMP1 and MMP3 expression in a similar manner between the two cell types. This data supports published reports [64,65] showing that TGF-β signaling increases MMP gene transcription. Our results suggest that an increase in basal expression of MMP1 and MMP3 may play a role in KC development; however, further work is warranted to determine if the altered ECM assembled by HKCs is primarily a result of ECM secretion, rather than degradation. Our data also suggests that activation of MMP expression via TGF-β stimulation following activation of the wound healing process may contribute to an increase in ECM degradation, which may be important in KC.

## 3. Experimental Section

### 3.1. Cell Culture

Corneas were obtained by the National Disease Research Interchange (NDRI) and processed as previously described [9,32,66]. Briefly, the endothelium and epithelium were removed by scraping briefly with a razor blade, they were then cut into ~ 2 mm × 2 mm pieces. The pieces of stroma were allowed to adhere to the bottom of a T75 flask for 30 minutes at 37 degrees Celsius before adding 10% Fetal Bovine Serum (FBS) Eagle’s Minimum Essential Media (EMEM) to the flask. After 2–4 weeks the explants were passaged in 10% FBS in EMEM.

### 3.2. Collagen Contraction Assay

Rat-tail Collagen type I (Advanced Biomatrix) was mixed with EMEM on ice with 125 μL EMEM per 1 mL Collagen. The pH was then adjusted to pH 7–8 with 1 M NaOH. HCFs or HKCs were added at a concentration of 5 × 10^5^ and mixed slowly to avoid air bubbles. This mixture was plated in a 12 well plate at 1 mL per well and incubated in 37 °C for 30 min to promote solidification. After congealing 1 mL of 10% FBS EMEM was added on top of the construct. The collagen matrix constructs were released after 48 h of incubation by running a sterile blade around the edges of the well. Contraction was measured every other day for 4 weeks starting at 24 h after the initial release. Treated media was supplemented with 0.1 ng/mL of TGF-β1, TGF-β2, or TGF-β3, and the area of the gel was quantified using ImageJ software following imaging by camera. Changes in contraction were measured from day 0 to day 26. The constructs were fully contracted by day 26, and we did not observe any reduction in gel area after day 26.

### 3.3. RT-PCR

Fully contracted constructs at day 26 were placed into 1 mL Trizol and incubated at 22 °C for 5 min. 200 μL chloroform was added before shaking vigorously and centrifuging for 15 min at 1200 rpm. The supernatant was further purified using the Ambion RNA kit (Life Technologies, Carlsbad, CA, USA), following the protocol given, with the RNA being dissolved in 30 μL RNase free water. The LVis plate (Clariostar, BMG Labtech, Ortenberg, Germany) was used to measure the concentration and purity of the extracted RNA. A 10% solution of cDNA was made with RNase free water to use for the PCR. While a ratio of 10:7 master mix to RNase free water was made along with 2 μL of a 10% cDNA sample solution and 1 μL of Taqman gene specific assay (Life Technologies) per well. This was quantified using mean cT values obtained from life technologies Real Time Thermal Cycler with standards conditions for Taqman gene expression probes (Applied Biosystems, Foster City, CA, USA) for 40 cycles. The following probes were purchased from Life Technologies: MMP1 (Hs00899658_m1), MMP3 (Hs00968305_m1), and Collagen I (Hs00164004_m1), Collagen III (Hs00943809_m1), and Collagen V (Hs00609133_m1). GAPDH (Hs99999905_m1) and 18S (Hs99999901_s1) probes were used as endogenous controls (Table 1).

**Table 1:** RT-PCR probes and their concentrations.

Probe	Catalogue #	Company	Final Concentration
GAPDH	Hs99999905_m1	Life Technologies	1×
18S	Hs99999901_s1	Life Technologies	1×
Col I	Hs00164004_m1	Life Technologies	1×
Col III	Hs00943809_m1	Life Technologies	1×
Col V	Hs00609133_m1	Life Technologies	1×
MMP 1	Hs00899658_m1	Life Technologies	1×
MMP 3	Hs00968305_m1	Life Technologies	1×

### 3.4. Western Blot

Western Blot was performed on media collected from the contracting matrix at 1 week. Total protein content within conditioned media was measured using a BCA assay (ThermoScientific, Rockford, IL, USA). Samples were then normalized to the sample containing the lowest protein content, thereby enabling equal loading onto the gel. Media samples were then run on a 4%–20% pre-cast polyacrylamide gradient gel at 130 V for 1.5 h then transferred to a nitrocellulose membrane on ice at 100 V for 1 h. The membrane was blocked in a 5% milk solution in Tris-buffered Solution with Tween20 for 1 h, then incubated overnight in a cold room with 1:1000 primary antibody. Antibodies used include: Collagen (ab34710; Abcam, Cambridge, MA, USA), Collagen III (ab7778; Abcam), Collagen V (ab94673; Abcam) (Table 2). After primary incubation, the membrane was washed for 5 min (3×) in Tris-buffered Solution with Tween20 before probing with secondary antibody Goat anti-Rb Alexafluor 568 (Life Technologies, Grand Island, NY, USA) at room temperature for 1 h with rocking. The membrane was allowed to dry before imaging using ChemiDoc-it to image. Western blots were quantified using densitometry utilizes pixels measured within each band.

**Table 2:** Western blot antibodies and final dilutions.

Antibody	Catalogue #	Company	Dilution
Col I	ab34710	Abcam, Cambridge, MA, USA	1/1000
Col III	ab7778	Abcam, Cambridge, MA, USA	1/1000
Col V	ab94673	Abcam, Cambridge, MA,USA	1/1000

### 3.5. Statistical Analysis

Statistical analyses were carried out using a two-way ANOVA test calculated by GraphPad Prism software. *p* < 0.05 were considered statistically significant. Error bars represent standard error of the mean. Data is representative of three independent experiments.

## 4. Conclusions

In this study, we found that HKCs have a significant reduction in initial contractility of the matrix and altered Collagen expression compared to HCFs. Contraction of the ECM is important in normal wound healing processes within the cornea [67,68]. The defect in contraction exhibited by HKCs suggests that stromal fibroblasts in KC corneas are less able to respond to external stimuli and have delayed closure of the surrounding matrix following wounding. The failure to respond properly to normal wound healing mechanisms following injury can cause significant pathologies within the cornea [69,70]. The role of eye rubbing in KC development has been posited [23], but has yet to be thoroughly explored as the causative agent of KC pathogenesis. Our study suggests that HKCs have reduced contractility and thereby are less able to perform normal wound closure within the cornea following trauma, which may occur following continual eye rubbing. Clearly, further work is warranted to identify the molecular defects present in HKCs that contribute to this phenotype. The TGF-β isoforms have been detected in the human tear film, with TGF-β1 as the dominant isoform [71]. In our study, we found that the TGF-β isoforms mediate accelerated contraction of the matrix by HKCs up to HCF levels supporting the potential role of altered TGF-β signaling in KC pathobiology. Moreover, HKCs exhibited lower Col I/Col III and Col I/Col V ratios compared to HCFs, suggesting a significant defect in collagen deposition by HKCs that may support a defected corneal stroma ECM. Our results show that MMP1, but not MMP3, was elevated in HKCs compared to HCFs suggesting that MMP1 may play a significant role in the KC pathology. Overall, our study identified novel defects in HKCs that give rise to altered ECM contractility and composition that may contribute to the pathological ECM present in KC. In future studies, we will identify the molecular mechanism supporting reduced contractility by HKCs and relate this data to the KC phenotype *in vivo*.
